# Surgical management of mucinous adenocarcinoma arising in perianal fistula: A case series

**DOI:** 10.1016/j.sopen.2025.04.005

**Published:** 2025-04-17

**Authors:** Saeed Derakhshani, Milad Karimian Ghadim, Abolfazl Salari, Mohammadreza Ghahari

**Affiliations:** a Department of General Surgery, School of Medicine, Shahid Beheshti University of Medical Sciences, Tehran, Iran; b Kasra General Hospital, Tehran, Iran; c Cancer Research Center, Cancer Institute of Iran, Tehran University of Medical Sciences, Tehran, Iran; d Research Center of Biomedical Technology and Robotics (RCBTR), Advanced Medical Technologies and Equipment Institute (AMTEI), Tehran University of Medical Sciences, Tehran, Iran; e Advanced Diagnostic and Interventional Radiology Research Center (ADIR), Tehran University of Medical Sciences, Tehran, Iran

**Keywords:** Mucinous adenocarcinoma, Abdominoperineal resection, Perianal fistula

## Abstract

Perianal mucinous adenocarcinoma (MAC) is a rare malignancy arising in the anal canal, often associated with chronic inflammatory conditions such as perianal fistulas. This case series aims to elucidate the clinical features and surgical management of patients with perianal MAC arising from perianal fistulas.

Four cases of perianal MAC are presented, highlighting the diverse clinical presentations, diagnostic pathways, and therapeutic interventions. Each case demonstrates unique aspects of disease progression, treatment response, and long-term outcomes. Key features include the challenges of diagnosing MAC amidst benign conditions, the role of neoadjuvant chemotherapy in improving resectability, and the importance of individualized surgical approaches.

All patients were male and presented with a variety of symptoms ranging from changes in bowel habits to perianal abscesses. The diagnosis was challenging due to the resemblance of MAC to benign conditions and delayed presentation. All patients underwent abdominoperineal resection (APR) and colostomy insertion (perineal or abdominal). Two cases required reconstructive procedures where a V—Y flap and an omental flap were employed. A multidisciplinary approach ensures that patients receive appropriate neoadjuvant and adjuvant treatment. With the median follow-up of 3 years, all patients were alive.

Our multidisciplinary approach effectively managed perianal MAC by integrating surgical techniques, including APR and reconstructive methods, along with neoadjuvant and adjuvant treatment. Performing appropriate surgical techniques leads to tumor-free margins, in addition to systemic therapy, and improves both patient survival and quality of life.

## Highlights

• Perianal mucinous adenocarcinoma (MAC) is a rare and aggressive malignancy that frequently mimics benign perianal diseases.
• Four cases of MAC arising in perianal fistula were treated with abdominoperineal resection, followed by V—Y flap if needed.
• With a median follow-up of 3 years, all patients were alive.
• Performing appropriate surgical techniques and perioperative systemic therapy improves patient survival.

## Graphical abstract

**Figure:**
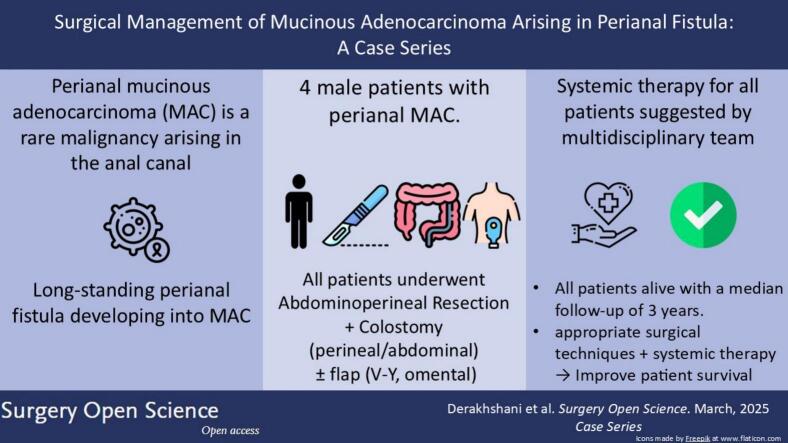

## Introduction

Perianal mucinous adenocarcinoma (MAC) is a rare and aggressive malignancy that arises in the lower third of the anal canal, accounting for less than 5 % of all anal tumors [1,2]. MAC is characterized by the infiltration of malignant glandular cells containing intracytoplasmic mucin. Factors such as recurrent friction, scarring, and inflammation have been implicated in the pathogenesis of perianal MAC [3,4]. Additionally, substantial evidence links inflammatory bowel disease (IBD) to the development of MAC [5,6]. It has been proposed that the transformation of a chronic perianal fistula into malignancy accounts for 3 % to 11 % of perianal cancers [7], and it could be associated with the regeneration of mucosal tissue. Usually, the invasive glandular formations correlate with the development of a stroma characterized by mucoid consistency [8]. Normally, in the shape of alveolar, row-like, or single-scattered cells. MAC frequently mimics benign perianal diseases such as abscesses or fistulas. This leads to delayed diagnosis, with many patients presenting at an advanced stage, further complicating treatment and adversely affecting prognosis [2,[9], [10], [11]].

This study aims to present four cases of perianal MAC arising from perianal fistulas. Patients' clinical features, diagnostic challenges, and treatment strategies are discussed. Also, it focuses on the significance of surgical treatment as a key component of the treatment plan while also focusing on perioperative treatments to improve outcomes of patients with this rare cancer.

## Case presentation

### Case 1

A 53-year-old male patient presented with altered bowel habits and rectal bleeding. Investigations revealed rectal cancer, and the patient subsequently underwent low anterior resection (LAR) with ileostomy. During surgery, a perianal fistula located near the rectal adenocarcinoma and anus was discovered, raising suspicion of malignancy. A biopsy was taken from the fistula tract, followed by seton placement. Pathological examination of the rectal specimen confirmed well-differentiated adenocarcinoma, staged as T3aN0, with wide tumor-free margins. Furthermore, the fistula biopsy confirmed a diagnosis of MAC. Therefore, neoadjuvant chemotherapy (NAC) was recommended by our multidisciplinary team.

Two months after NAC, the patient underwent an abdominoperineal resection (APR), perineal colostomy, and an antegrade colonic enema (ACE). However, the patient refused the placement of an ileostomy, so we closed it during a subsequent surgery. The pathology report revealed a T2N0 tumor with no margin involvement (Table 1). The patient declined the use of an abdominal colostomy bag due to personal and cultural preferences. To address these concerns, we chose an alternative approach. In this procedure, the colon was sutured to the perineum, where the anus is located, instead of using a permanent abdominal colostomy bag. In order to manage fecal incontinence, we performed an ACE procedure. During the procedure, the surgeon created an appendicostomy by connecting the end of the appendix to the cecum. The appendicostomy was created small enough that it would have minimal impact on the patient's body image. Additionally, the ostomy was created to prevent leakage, with the few secretions that would soil the patient's clothing.

**Table 1:** Baseline characteristics of patients, systemic and surgical treatments, and follow-up.

Subject/Patient	Case 1	Case 2	Case 3	Case 4
Age	53	62	65	67
Sex	Male	Male	Male	Male
Tumor TNM Stage	T2N0	T3N0	T3N0	T3N0
Systemic Treatment	Neoadjuvant and adjuvant therapy	Neoadjuvant and adjuvant therapy	Neoadjuvant and Adjuvant therapy	Neoadjuvant and adjuvant therapy
Surgical Treatment	APR (perineal colostomy, ACE)	APR (perineal colostomy, ACE, bilateral V—Y flap)	APR (abdominal colostomy)	APR (abdominal colostomy, bilateral V—Y flap, omental flap)
Recurrence	+	−	−	−
Duration of Follow-up	4 years	2 years	4 years	1 year
Living Status	Alive	Alive	Alive	Alive

APR: abdominoperineal resection; ACE: antegrade colonic enema.

This procedure allows the patient to perform a complete colonic washout by flushing stool from the colon downward after inserting a water enema via a catheter. The patient was educated on how to irrigate the colon daily using one liter of water. This is likely to improve the patient's quality of life, enabling them to resume normal activities. This approach not only addresses the patient's medical needs but also preserves their body image and cultural sensitivities. Furthermore, the patient may experience reduced or absent soiling of undergarments.

After surgery, the patient underwent adjuvant chemotherapy (AC). Twenty-nine months after surgery, a follow-up evaluation for recurrence of the tumor included a CT scan that demonstrated a distant metastatic nodule at the site where the perineal colostomy was embedded in the skin between the perineum and the colon. The pathology analysis confirmed that the metastatic nodule was adenocarcinoma related to MAC in a perianal fistula. Treatment included chemotherapy and local excision of the nodule. During a four-year follow-up period after diagnosis, no evidence of local recurrence or further metastases was observed, and the patient remains alive.

### Case 2

A 62-year-old male patient with a history of multiple surgeries for recurrent perianal fistula, with an abscess and pus discharge, was examined under anesthesia. During the examination, the fistula appeared elongated, and physical examination revealed another deformity: a hard and fixed mass occupying half of the posterior of the lower rectum. The result of a biopsy from the fistula tract confirmed the diagnosis of MAC.

**Fig. 1:**
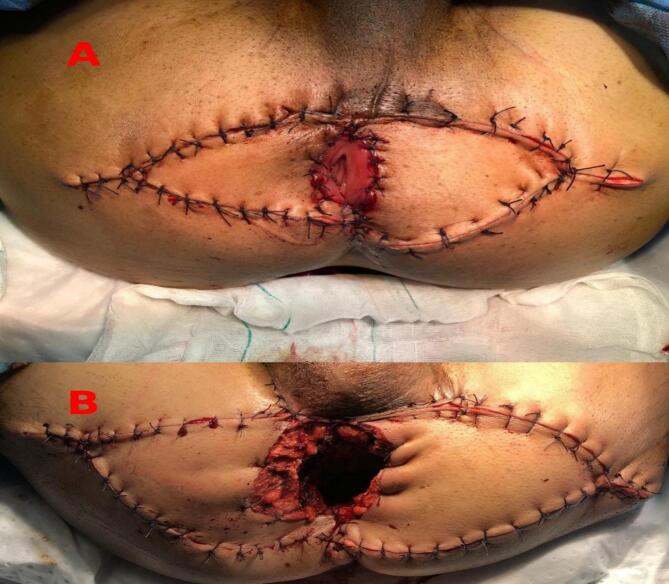
**1 A:** case 2 after abdominoperineal resection (APR), perineal colostomy, and reconstruction by bilateral V—Y flap; **1B:** case 4 after APR, abdominal colostomy, reconstruction by bilateral V—Y flap and omental flap.

Neoadjuvant chemoradiotherapy was performed. Subsequently, he underwent APR, perineal colostomy, and ACE surgery. However, according to the size of the tissue yield to be resected, we performed a V—Y flap technique on both sides of the gluteal area to cover the defect caused by APR, taking into account the size of the defect (Fig. 1A). The V—Y flap has certain benefits, such as a robust blood supply and consistent healing. Histological findings confirmed the mucinous type of adenocarcinoma (T3N0) with free-tumor surgical margins, indicating the procedure was successful. Subsequently, the individual received AC, and after two years of follow-up, there was no evidence of recurrence, and the patient remains alive.

### Case 3

A 65-year-old male with a notable medical history of sigmoid colon adenocarcinoma, previously treated with a sigmoidectomy, returned for a follow-up visit. A perianal fistula was identified during this visit, distinguished by its unusual stony texture. A biopsy was promptly performed on the fistula, revealing MAC within its pathology. Given the patient's medical background and the new diagnostic findings, neoadjuvant chemoradiotherapy was recommended.

Following chemoradiotherapy, the patient underwent an APR, succeeded by the construction of an abdominal colostomy for fecal diversion. The pathology report confirmed a T3N0 tumor without margin involvement. The patient's postoperative phase was uneventful, with satisfactory wound healing. Subsequently, the patient received AC to target any residual cancer cells and reduce the risk of recurrence.

Throughout the follow-up period of 4 years, no evidence of local recurrence or distant metastases was detected, and the patient is still alive.

### Case 4

A 67-year-old man complained of anal pain. After examination, a perianal abscess was diagnosed, and the patient underwent abscess drainage. Physical examination after the drainage revealed an unusual hardness in the area, prompting a recommendation for a colonoscopy. During the colonoscopy, a large submucosal lesion in the anus with extrinsic pressure on the anal canal, causing partial obstruction, was observed. Otherwise, the other parts of the colon were normal. A rectal mucosal biopsy confirmed MAC associated with a perianal fistula. We proposed that the patient undergo Neoadjuvant treatment, followed by surgery. After about 4 months of preoperative therapy, the patient underwent APR. Due to the extensive perineal excision, both a bilateral V—Y flap and an omental flap with vascular support were utilized to cover the resected area (Fig. 1B). However, the uncovered parts of the resection site were left open for secondary healing. Additionally, a colostomy was performed in an abdominal form at the patient's request. The pathology result revealed poorly differentiated MAC (T3aN0) without margin involvement. Sessions of AC were also prescribed.

Throughout the one-year follow-up period, no evidence of local recurrence or distant metastases was detected, and the patient is still alive.

## Discussion

Our case series highlights the unusual presentation of a long-standing perianal fistula developing into MAC, an uncommon occurrence in itself [12], and emphasizes how the absence of typical symptoms of MAC can contribute to delayed diagnosis and worsened prognosis [13]. Studies indicate that rectal bleeding and mucous discharge are the most frequent presenting symptoms of MAC, reported in up to 70 % of patients [14,15]. Out of the 4 patients we presented, 2 (50 %) experienced such symptoms.

Perianal fistulas, although common, rarely progress to malignant lesions, making diagnosis both challenging and often delayed. This underscores the importance of maintaining a high index of suspicion for malignancy, especially in patients with chronic perianal fistulas that exhibit changes in size, shape, or surrounding tissue characteristics [16].

As demonstrated in our cases, a comprehensive histological examination of biopsy samples is crucial for precise diagnosis, especially when atypical features are noted during physical examination. Biopsies might not always capture the infiltrative nature of the carcinoma [17,18], but it is essential to emphasize that histological diagnosis remains the definitive standard for accurate determination, thus advocating for the implementation of biopsy procedures to confirm the presence of well-differentiated (WD) MAC.

WD MAC is a rare variant of colorectal MAC characterized by low-grade nuclear atypia. It is considered a low-grade adenocarcinoma and grows slowly. When matched for T classification and tumor location, WD MAC shows lower rates of microsatellite instability and higher rates of chromosomal instability than MAC [19]. Since anal fistula occurs in 20 % of patients with Crohn's disease and the association of MAC and Crohn's disease has been widely established, further colorectal investigation is needed to rule out Crohn's disease in these patients [5,17].

The pathogenesis of perianal MAC remains unclear, with several factors implicated, including chronic inflammation, perianal abscesses, perianal Crohn's disease, and genetic mutations. A notable aspect of fistula-associated mucinous adenocarcinoma (FAMC) is its association with Crohn's disease (CD), which is considered a significant risk factor for this rare adenocarcinoma. Sjödahl et al. reported a 0.7 % incidence of perianal MAC development in patients with CD [20]. However, only one of our cases had a history of CD or other bowel habit changes, which emphasizes the heterogeneity in the etiological background of FAMC. Also, cryptoglandular infections attributing to 90 % of anal fistulas, the infectious theory posits a continuum from cryptitis to perianal abscess and subsequently anal fistula as phases of the same infectious condition, half of our cases had abscess contributed to the fistula. Further research is needed to elucidate the underlying mechanisms driving malignant transformation in perianal fistulas, which may aid in early detection and targeted interventions.

Optimal management of perianal MAC involves a multidisciplinary approach, including neoadjuvant therapy, surgical resection, and adjuvant therapy [3,[21], [22], [23], [24], [25], [26], [27], [28], [29]] with significantly improved outcomes [13]. The main treatment for perianal MAC is APR and wide local resection to achieve margin-free status. In patients with large defects, reconstructive surgery is needed. Furthermore, neoadjuvant treatment helps reduce tumor burden and improve resectability. Adjuvant therapy can also help eliminate any residual cancer cells, if present [30].

In our study, all cases underwent APR, followed by reconstructive surgery using the V—Y flap method as needed. Abdominal or perineal colostomy was performed based on patient preferences and feasibility. Furthermore, potential complications that the patient might face in the future were taken into account, and we conducted an ACE, creating an ostomy with the appendix into the cecum, to prevent fecal incontinence and improve the patient's quality of life. Additionally, a multidisciplinary approach ensured that our patients received neoadjuvant and adjuvant treatment properly.

Our study highlighted that performing surgery using appropriate techniques, achieving tumor-free margins, and providing systemic therapy improves both patient survival and quality of life. All of our patients were alive at the last follow-up, with only one patient experiencing recurrence. Our median follow-up period was three years, which exceeds previous studies demonstrating an 18-month survival for MAC patients treated with surgery alone [31]. This finding accentuates that systemic treatment is crucial in managing MAC; however, further studies are needed to clarify this important point.

## Conclusion

Our multimodal approach effectively managed perianal MAC by integrating neoadjuvant treatment, APR, V—Y flap reconstruction, and adjuvant treatment. Tailored treatments addressed both the malignancy and associated comorbidities, leading to notable improvements in functional status, symptom relief, and overall quality of life.

## CRediT authorship contribution statement

**Saeed Derakhshani:** Writing – review & editing, Writing – original draft, Investigation, Data curation. **Milad Karimian Ghadim:** Writing – review & editing, Supervision, Project administration, Investigation. **Abolfazl Salari:** Writing – original draft, Investigation. **Mohammadreza Ghahari:** Writing – original draft, Investigation.

## Ethical approval

This study doesn't include any personal information of patients that could lead to their identification. Therefore, this study is exempt from ethical approval.

## Funding

This research did not receive any specific grant from funding agencies in the public, commercial, or not-for-profit sectors.

## Declaration of competing interest

The authors declare no conflicts of interest relevant to this article.
